# Hepatitis B virus-associated diffuse large B-cell lymphoma: unique clinical features, poor outcome, and hepatitis B surface antigen-driven origin

**DOI:** 10.18632/oncotarget.4677

**Published:** 2015-07-22

**Authors:** Lijuan Deng, Yuqin Song, Ken H. Young, Shimin Hu, Ning Ding, Weiwei Song, Xianghong Li, Yunfei Shi, Huiying Huang, Weiping Liu, Wen Zheng, Xiaopei Wang, Yan Xie, Ningjing Lin, Meifeng Tu, Lingyan Ping, Zhitao Ying, Chen Zhang, Yingli Sun, Jun Zhu

**Affiliations:** ^1^ Key Laboratory of Carcinogenesis and Translational Research (Ministry of Education), Lymphoma Unit, Peking University Cancer Hospital & Institute, Beijing, China; ^2^ Department of Hematopathology, The University of Texas MD Anderson Cancer Center, Houston, TX, USA; ^3^ Department of Pathology, Peking University Cancer Hospital & Institute, Beijing, China

**Keywords:** hepatitis B virus, diffuse large B-cell lymphoma, hepatitis B surface antigen, B-cell receptor, complementarity determining region 3

## Abstract

While the epidemiologic association between hepatitis B virus (HBV) infection and diffuse large B-cell lymphoma (DLBCL) is established, little is known more than this epidemiologic evidence. We studied a cohort of 587 patients with DLBCL for HBV infection status, clinicopathologic features, and the immunoglobulin variable region in HBV surface antigen (HBsAg)-positive patients. Eighty-one (81/587, 13.8%) patients were HBsAg-positive. Compared with HBsAg-negative DLBCL, HBsAg-positive DLBCL displayed a younger median onset age (45 vs. 55 years), more frequent involvement of spleen or retroperitoneal lymph node (40.7% vs. 16.0% and 61.7% vs. 31.0% respectively, both *p* < 0.001), more advanced disease (stage III/IV: 76.5% vs 59.5%, *p* = 0.003), and significantly worse outcome (2-year overall survival: 47% versus 70%, *p* < 0.001). In HBsAg-positive DLBCL patients, almost all (45/47, 96%) amino acid sequences of heavy and light chain complementarity determining region 3 exhibited a high homology to antibodies specific for HBsAg, and the majority (45/50, 90%) of *IgHV* and *IgLV* genes were mutated. We conclude that 13.8% of DLBCL cases are HBV-associated in HBV-endemic China and show unique clinical features and poor outcomes. Furthermore, our study strongly suggests that HBV-associated DLBCL might arise from HBV antigen-selected B cells.

## INTRODUCTION

As early as in 1970s, the detection of hepatitis B surface antigen (HBsAg) in the hepatocytes of some patients with lymphoproliferative disorders was reported and first suggested this association [1, 2]. Since then, an epidemiologic association between HBV infection and non-Hodgkin's lymphoma (NHL) was confirmed by a large number of retrospective or prospective, case-control or cohort studies, as well as two meta-analyses [3–9]. A most recent meta-analysis, in which over 40,000 cases of NHL and 1,660,000 cases of control were included, showed that HBV-infected individuals had an odd ratio of 2.24 (95% confidence interval 1.80–2.78; *P* ≤ 0.001) of developing NHL [9]. With regard to the NHL subtypes, a firm association was found with B-cell NHL, especially with diffuse large B-cell lymphoma (DLBCL), the most common subtype of B-cell NHL, but not with T-cell NHL [9]. In HBV prevalent countries, the odd ratio of DLBCL in HBV-infected individuals was 2.73 (95% confidence interval 1.62–4.59; *P* ≤ 0.001) [9].

Such an association between HBV and NHL would parallel that between another subtype of hepatitis virus, hepatitis C virus (HCV) and NHL [10, 11]. With a histologic predominance of DLBCL and marginal-zone lymphoma, HCV-associated lymphomas often involve spleen or target organs of HCV infection such as liver and salivary glands [10, 11]. Possible mechanisms of HCV-induced lymphoma-genesis are chronic viral antigen stimulation, an interaction between HCV-E2 and CD81 expressed on B-cells, and direct HCV infection of B-cells. Among these putative mechanisms, chronic viral antigen stimulation is the most plausible mechanism [10, 11].

Ironically, although HBV infection is more prevalent than HCV, other than the epidemiologic association, little is known regarding both clinical features and pathogenesis of HBV-associated lymphoma [12]. Because the anti-B-cell therapy with rituximab has increased the risk of HBV reactivation, the prophylaxis and management of this complication has become a major issue in the management of B-cell lymphoma patients with chronic HBV infection [13]. In contrast, much less attention has been paid to the lymphoma itself in these patients. The treatment for HBsAg-positive DLBCL patients is largely similar to that for HBsAg-negative DLBCL patients except additional antiviral prophylaxis. Both the clinicopathologic features of HBV-associated lymphoma and the possible pathogenic mechanism of HBV in lymphoma remain largely unclear [10].

China is an endemic area for the HBV infection with a HBsAg-positive rate of 7.18% in the general population and about 93 million chronically-infected patients [14, 15]. The high prevalence of HBV infection could represent a unique opportunity to study the association between HBV and lymphoma [9]. By retrospectively analyzing the clinical data of a cohort of 587 patients with DLBCL, we found HBsAg-positive DLBCL patients presented unique clinical features and had significantly poor outcome. Our further analysis of immunoglobulin (Ig) heavy and light chain variable region genes in HBsAg-positive DLBCL patients subsequently strongly suggests that HBV-associated DLBCL might arise from HBV antigen-selected B cells.

## RESULTS

### Baseline characteristics

The baseline clinical characteristics of the 587 patients are shown in Table 1. Eighty-one patients were HBsAg-positive (81/587, 13.8%) and 506 (86.2%) were HBsAg-negative. Of HBsAg-negative patients, 8 were HCV-positive. All patients were HIV-negative. Thirty-three of the 81 (40.7%) HBsAg-positive and 271 of the 506 (53.6%) HBsAg-negative DLBCL patients were treated with R-CHOP and others were treated with CHOP. The median follow-up time was 41 months (range 1–135) for the whole group, and was 27 months (range 1–116) for HBsAg-positive group and 43 months for HBsAg-negative group (range 1–135).

**Table 1:** Clinical characteristics of HBsAg-positive and negative patients

Clinical factor	HBsAg-positite patients (*n* = 81) n (%)	HBsAg-negative patients (*n* = 506) n (%)	*P* value
Special sites involvement			
Liver	9 (11.1)	41 (8.1)	0.368
Spleen	33 (40.7)	81 (16.0)	**<0.001**
Bone	2 (2.5)	19 (3.8)	0.563
Stomach	11 (13.6)	88 (17.4)	0.395
Intestinal	9 (11.1)	53 (10.5)	0.827
Tonsil	10 (12.3)	50 (9.9)	0.497
Bone marrow	3 (3.7)	15 (3.0)	0.720
Retroperitoneal lymph node	50 (61.7)	157 (31.0)	**<0.001**
Age > 60	12 (14.8)	187 (37.0)	**<0.001**
Stage III/IV	62 (76.5)	301 (59.5)	**0.003**
Gender, male	51 (63.0)	273 (54.0)	0.122
B symptom	47 (58.0)	183 (36.2)	**<0.001**
Performance status 2–4	10 (12.3)	57 (11.3)	0.777
IPI 3–5	34 (42.0)	155 (30.6)	**0.043**
Elevated LDH	47 (58.0)	205 (40.5)	**0.003**
Bulky mass	13 (16.0)	71 (14.0)	0.909
Extra-nodal sites ≥ 2	36 (44.4)	178 (35.2)	0.133
Use of rituximab	33 (40.7)	271 (53.6)	**0.032**
Response to primary chemotherapy			**<0.001**
Complete response	30 (37.0)	326 (64.4)	
Partial response	15 (18.5)	84 (16.6)	
Stable disease	1 (1.2)	5 (1.0)	
Progressive disease	35 (43.2)	81 (16.0)	
Not available	0 (0.0)	10 (2.0)	

Numbers in bold are statistically significant. IPI, International Prognostic Index; LDH, lactate dehydrogenase.

Bulky mass ≥ 10 cm.

### HBV infection status and unique clinical features of HBsAg-positive DLBCL patients

The HBV infection status of those 81 HBsAg-positive DLBCL patients were classified into three groups based on the viral markers including HBV DNA loads, the alanine transaminase and aspartate transaminase levels, and the ultrasonography or computerized tomography (CT) of liver [16]. The majority (56/81, 69.1%) were HBV carriers; ten patients had chronic hepatitis B; and 15 patients were diagnosed as hepatitis B cirrhosis, 13 (86.7%) of which were compensated cirrhosis. Forty-eight of the 81 (59.3%) patients had a documented history of chronic HBV infection and half of them have had disease for more than 20 years. Only one of the 242 HBsAg-negative and HBcAb-positive patients was positive for HBeAg.

**Figure 1:**
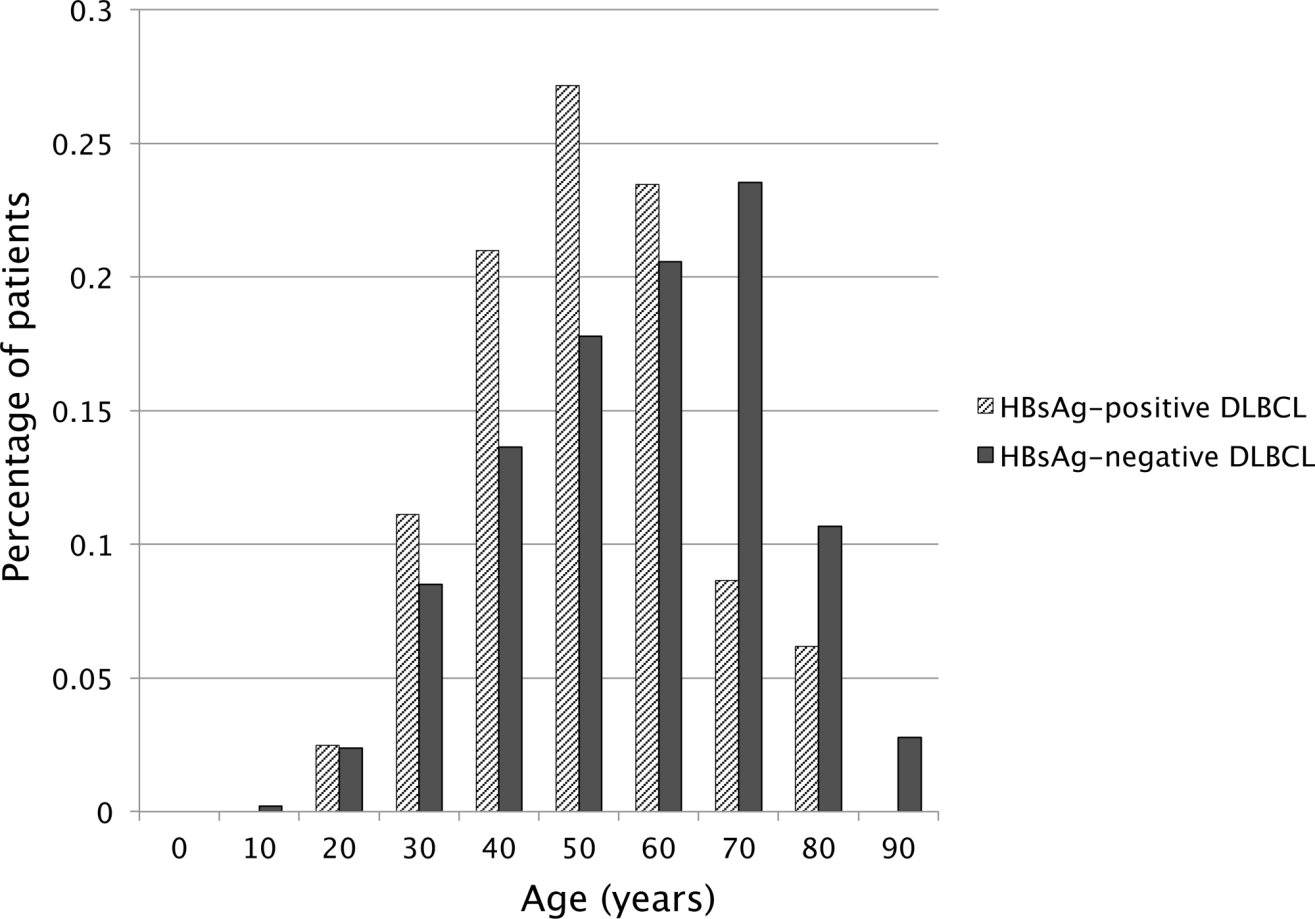
Age distribution of DLBCL patients in HBsAg-positive and HBsAg-negative groups

Compared with patients in HBsAg-negative group, patients in HBsAg-positive group displayed younger age with a median age of 45 years (range 16–78) vs 55 years (range 9–90) in the HBsAg-negative group (Figure 1). The majority of patients in the HBsAg-positive group were in the age from 36 to 55 years (54.3% versus 34.0%) and fewer were over 55 years (25.9% versus 48.0%). DLBCL in HBsAg-positive group showed more frequent involvement of spleen or retroperitoneal lymph node (40.7% vs. 16.0% and 61.7% vs. 31.0% respectively, both *p* < 0.001), more advanced disease (stage III/IV: 76.5% vs 59.5%, *p* = 0.003), B symptoms (58.0% vs 36.2%, *p* < 0.001), high International Prognostic Index (IPI) (IPI ≥ 3: 42.0% vs 30.6%, *p* = 0.043) and elevated lactate dehydrogenase (LDH: 58.0% vs 40.5%, *p* = 0.003).

### Poor outcome of HBsAg-positive DLBCL patients

**Figure 2:**
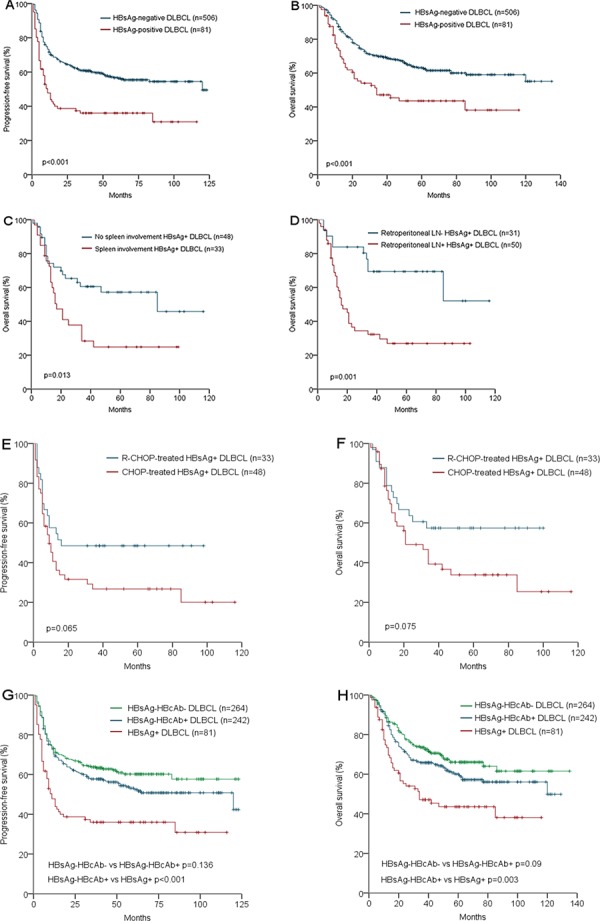
Survival analysis according to HBV infection status Progression-free survival of HBsAg-positive and HBsAg-negative DLBCL patients **A.** Overall survival of HBsAg-positive and HBsAg-negative DLBCL patients **B.** Overall survival of HBsAg-positive DLBCL patients with and without spleen involvement **C.** Overall survival of HBsAg-positive DLBCL patients with and without retroperitoneal LN involvement **D.** Progression-free survival of HBsAg-Positive DLBCL patients treated with R-CHOP and CHOP regimen **E.** Overall survival of HBsAg-Positive DLBCL patients treated with R-CHOP and CHOP regimen **F.** Progression-free survival of HBsAg+, HBsAg-HBcAb+, and HBsAg-HBcAb- DLBCL patients **G.** Overall survival of HBsAg+, HBsAg-HBcAb+, and HBsAg-HBcAb- DLBCL patients **H.**

Patients in HBsAg-positive group showed significantly worse OS and PFS than those in the HBsAg-negative group with 2-year OS of 47% vs 70% and 2-year PFS of 36% vs 61% (both *p* < 0.001, Figure 2A and 2B), and 5-year OS of 44% vs 61% and 5-year PFS of 36% vs 55% ( *p* = 0.012 and 0.005 respectively). For HBsAg-positive DLBCL patients, once spleen or retroperitoneal lymph node were involved, the 2-year survival was lower than 40% (Figure 2C and 2D). In HBsAg-positive DLBCL group (Table 2), there was a strong trend that the response in the R-CHOP group was better than that in the CHOP group with a higher rate of complete response (57.6% vs 22.9%, *p* = 0.051). However, progressive disease (PD) occurred in about 40% of the patients in both groups. Moreover, 59.6% of the PD occurred within 6 months and 82.4% occurred within 12 months after the initiation of the primary therapy. Correspondingly, there was a trend toward a better PFS and the OS in the HBsAg-positive DLBCL patients treated with R-CHOP compared with those with CHOP (Figure 2E and 2F, *p* = 0.065 and 0.075, respectively). By analysis, 44 of the 81 HBsAg-positive patients died and the only cause of death was lymphoma; 180 of the 506 HBsAg-negative patients died, 164 of them died from lymphoma and the other 16 patients died from either therapy-related complications or other causes.

**Table 2:** Treatment response in HBsAg-positive patients

	R-CHOP (*n* = 33) *n* (%)	CHOP (*n* = 48) *n* (%)	*P* value
Response			0.051
Complete response	19 (57.6)	11 (22.9)	
Partial response	1 (3.0)	14 (29.2)	
Stable disease	0 (0)	1 (2.1)	
Progressive disease	13 (39.4)	22 (45.8)	

Abbreviations: CHOP, cyclophosphamide, doxorubicin, vincristine, and predinisone; R-CHOP, addition of rituximab to CHOP.

Hepatic dysfunction higher than Common Terminology Criteria for Adverse Events v4.0 (CTCAE4.0) grade 2 was only observed in 5 of 81 (6.2%) HBsAg-positive DLBCL patients. HBV reactivation occurred in another 5 of 81 (6.2%) HBsAg-positive DLBCL patients. All the hepatic dysfunction and HBV reactivation was under control. When HBsAg-negative DLBCL patients were further categorized as HBcAb-positive and HBcAb-negative groups, there was a trend toward a better PFS and OS in HBcAb-negative patients than HBcAb-positive patients (*p* = 0.136 and 0.09 respectively, Figure 2G and 2H). HBV-DNA was tested in 60 of the 81 HBsAg-positive patients. HBV-DNA was positive in 36 patients (median 3.6 * 10^6^, range: 2 * 10^2^ to 1.2 * 10^9^ IU/ml) and negative in 24 patients. In further survival analysis, both PFS and OS showed no difference between HBV-DNA-positive and HBV-DNA-negative patients.

### Cell of origin and outcome in HBsAg-positive DLBCL patients

Thirty-three cases (33/49, 67.3%) in HBsAg-positive group compared with 53 (53/93, 56.9%) in HBsAg-negative and HBcAb-negative group were classified as non-germinal center B-cell (non-GCB)-like respectively. There was no significant difference in the percentage of non-GCB between these two groups (* p* = 0.24). For the 49 HBsAg-positive DLBCL patients, PFS and OS did not differ between the GCB and non-GCB subtypes (Supplemental Figure 1A and 1B, *p* = 0.956 and 0.945, respectively).

### Biased immunoglobulin gene usage and mutated genotype

Total DNA was extracted from the FFPE tumor tissue of 47 cases of HBsAg-postitive DLBCL. Complete *IGHV-D-J* rearrangements were identified in 19 of 47 patients. Of those, 16 were potential productive rearrangements whereas 3 were nonproductive. A total of 29 *IGKV-J* rearrangements were detected and all of them were productive. Among them, two cases carried both potentially functional *IGKV-J* and *IGLV-J* rearrangements. Nonfunctional *IGLV-J* rearrangements were detected in another two patients.

As shown in Table 3 and Table 4, the most frequent *IGHV* gene was *IGHV4-34* (8/19, 42.1%) in our cohort. In the remaining 11 cases, 6 cases (31.6%) used *IGHV1* and 5 (26.3%) used *IGHV3* family genes. A stronger bias at the level of *IGKV* gene usage was observed with *IGKV4-1* in 19/29 (65.5%) cases. As shown in Table 3 and Table 4, 13 of 19 (68.4%) *IGHV* genes were highly mutated, 3 (15.8%) minimally/borderlinely mutated, and 3 (15.8%) truly unmutated. Except two minimally/borderlinely mutated *IGKV* sequences, all the *IGKV* and *IGLV* sequences were highly mutated.

**Table 3:** Ig heavy chain variable region analysis in HBsAg-positive DLBCL

No.	IgH CDR3 AA sequences	CDR3 AALength	Sequences homology	E value	VH	DH	JH	Identity to germline gene
10	CAKHEENSGWIFDNW-------	13	HBsAb heavy chain	0.67/6.3	IGHV3-23	IGHD5-12	IGHJ4	93.88%
30	CAKHEENSGWIFDNW-------	13	HBsAb heavy chain	0.67/6.3	IGHV3-23	IGHD5-12	IGHJ4	93.88%
11	CARAGETATTPGRGAFDIW-------	17	HBcAb heavy chain	18	IGHV1-18	IGHD5-24	IGHJ3	90.48%
12	CARDGRTAVTNPFDYW-------	14	HBsAb heavy chain	0.005	IGHV1-24	IGHD4-17	IGHJ4	97.87%
4	CARGDTSGPSDFW-------	11	HBsAb heavy chain	367	IGHV4-34	IGHD3-22	IGHJ4	87.50%
28	CARGEIVVVPAAAYYYYYMDVW	20	HBsAb heavy chain	2e-06/5e-04	IGHV4-34	IGHD2-2	IGHJ6	97.44%
13	CARGGLESTAGFFWFDPW------- .	16	HBsAb heavy chain	2e-07/1e-05	IGHV4-34	IGHD1-26	IGHJ5	97.50%
27	CARVEERYFYESSGYFDYW-------	17	HBsAb heavy chain	0.008	IGHV4-34	IGHD3-22	IGHJ4	92.50%
5	CGSTSSPWLYLGGMDVW-------	15	HBsAg binding protein	0.038	IGHV1-18	IGHD2-15	IGHJ6	93.62%
31	CVKGGLWFGVYDYYGMDVW---	17	HBsAg binding protein	8e-05	IGHV3-11	IGHD3-10	IGHJ6	100.00%
32	CVKGGLWFGVYDYYGMDVW---	17	HBsAg binding protein	8e-05	IGHV3-11	IGHD3-10	IGHJ6	100.00%
9	CVRAGYYYESTGFLYYFDYW---	18	HBsAb heavy chain	0.003	IGHV1-45	IGHD3-22	IGHJ4	95.74%
33	CVRDFFGDDSSIRDNCFDPW----	18	HBsAb heavy chain	7.3/7.6	IGHV4-34	IGHD2-21	IGHJ5	95.00%
14	CVRGSSSGFWGDLRSGYFDSW---	19	HBsAb heavy chain	0.016/2.1	IGHV1-24	IGHD2-15	IGHJ4	93.62%
34	CVRHPYDSDGPYYYYGMDVW---	18	HBsAg binding protein	4e-06	IGHV4-34	IGHD3-22	IGHJ6	95.00%
35	CVRLDYSNGWFDSW-------	12	HBsAb heavy chain	0.33/8.4	IGHV4-34	IGHD1-20	IGHJ5	95.00%
36	NA	NA	NA	NA	IGHV1-24	IGHD1-14	IGHJ4	100.00%
29	NA	NA	NA	NA	IGHV3-11	IGHD2-21	IGHJ4	95.92%
2	NA	NA	NA	NA	IGHV4-34	IGHD4-17	IGHJ5	92.50%

Abbreviations: IgH, immunoglobulin heavy chain; CDR3 AA, complementarity determining region 3 amino acid sequence.

**Table 4:** Ig light chain variable region analysis in HbsAg-positive DLBCL

No.	IgL CDR3 AA sequences	CDR3 AA Length	Sequences homology	E value	VL	JL	Identity to germline gene
1	CMQDTHWPP---	7	Ig Kappa chain	4e-07	IGKV2-30	IGKJ1	98.75%
2	CQQYNSYPLTF-	9	HBsAb	0.041	IGKV1-13	IGKJ2	64.91%
3	CLQHSEYPFTF-	9	HBsAb	5e-04	IGKV1-17	IGKJ3	92.16%
4	CTQATQFPYTF-	9	HBsAb	0.2/0.2	IGKV2-24	IGKJ2	99.17%
5	CQHYAEWPWTF-	9	HBsAb	1e-04	IGKV3-15	IGKJ1	74.65%
6	CQQYGDSPLTF-	9	HBsAb	0.078	IGKV3-20	IGKJ5	90.35%
7	CQQYFTPPRTF-	9	HBsAb	2e-05	IGKV4-1	IGKJ4	75.93%
8	CQQYYSIPLTF-	9	HBsAb	3e-05	IGKV4-1	IGKJ4	97.01%
9	CQQYYSIPLTF-	9	HBsAb	2e-08	IGKV4-1	IGKJ4	97.01%
10	CQQYFSNPLTF-	9	HBsAb	3e-07	IGKV4-1	IGKJ4	96.58%
11	CQQYFSNPLTF-	9	HBsAb	3e-07	IGKV4-1	IGKJ4	96.58%
12	CQQYFSNPLTF-	9	HBsAb	3e-07	IGKV4-1	IGKJ4	96.58%
13	CQQYFSNPLTF-	9	HBsAb	3e-07	IGKV4-1	IGKJ4	96.15%
14	CQQYFSNPLTF-	9	HBsAb	3e-07	IGKV4-1	IGKJ4	96.58%
15	CQQYFSNPLTF-	9	HBsAb	3e-07	IGKV4-1	IGKJ4	96.54%
16	CQQYFSNPLTF-	9	HBsAb	3e-07	IGKV4-1	IGKJ4	96.58%
17	CQQYFSNPLTF-	9	HBsAb	3e-07	IGKV4-1	IGKJ4	96.58%
18	CQQYFSNPLTF-	9	HBsAb	3e-07	IGKV4-1	IGKJ4	96.58%
19	CQQYFSNPLTF-	9	HBsAb	3e-07	IGKV4-1	IGKJ4	96.15%
20	CQQYFSNPLTF-	9	HBsAb	3e-07	IGKV4-1	IGKJ4	96.58%
21	CQQYFSNPLTF-	9	HBsAb	3e-07	IGKV4-1	IGKJ4	96.58%
22	CQQYFSNPLTF-	9	HBsAb	3e-07	IGKV4-1	IGKJ4	96.58%
23	CQQYFSNPLTF-	9	HBsAb	7e-06/4e-04	IGKV4-1	IGKJ4	96.58%
24	CQQYYTTPRTF-	9	HBsAb	5e-04/0.005	IGKV4-1	IGKJ4	96.58%
25	CQQYYTTPRTF-	9	HBsAb	5e-04/0.005	IGKV4-1	IGKJ4	96.58%
26	CQQNYSYPLTF-	9	HBsAb	4e-04/0.03	IGKV1-8	IGKJ4	96.30%
	CYSYTRHAAWVF	10	HBsAb	24/126	IGLV2-5	IGLJ3	89.00%
27	GQRTYNAPYTF-	9	HBsAb	1197/1197	IGKV1–37	IGKJ2	97.06%
	CQAWDIGTGVF-	9	HBsAb	792	IGLV3–1	IGLJ1	95.50%
28	NA	NA	NA	NA	IGLV3–19	IGLJ3	69.91%
29	NA	NA	NA	NA	IGLV3–27	IGLJ2	93.81%

Abbreviations: IgL, immunoglobulin light chain; CDR3 AA, complementarity determining region 3 amino acid sequence.

### Stereotyped CDR3 sequences highly homologous to HBsAbs

According to previously described criteria to define a stereotyped CDR3 sequence, two cases had an identical heavy chain CDR3 (HCDR3) sequence and another two cases had another identical HCDR3 sequence. The HCDR3 sequence in the remaining 12 cases did not satisfy the criteria for stereotype. Interestingly, 19 of 27 (70.5%) kappa chain CDR3 (KCDR3) sequences were highly homologous and met the criteria for stereotyped rearrangements and 14 of the 19 sequences were identical. A specific combination of *IGKV4-1* and *IGKJ4* usage was also observed in these 19 patients.

Notably, the HCDR3 sequences exhibited a high homology to antibodies specific for HBsAg in 15 of 16 patients, and for HBcAg in the remaining one patient. Furthermore, the KCDR3 sequences in 28 of 29 patients (96.6%) and the lambda chain CDR3 (LCDR3) sequences in 2 patients also showed the highest homology to antibodies specific for HBsAg. In 9 HBsAg-positive DLBCL patients, both heavy and light chain CDR3 sequences were obtained and all presented a high homology to HBsAb.

## DISCUSSION

In this study, we analyzed a consecutive cohort of 587 DLBCL patients from China, a HBV prevalent area. Our study showed that HBsAg-positive patients accounted for 13.8% of the whole cohort and might be a unique subset of DLBCL based on its clinical features and poor outcome. Additionally, we provide evidence for the first time that chronic HBV-associated antigen stimulation might play an important role in the pathogenesis of this subset of DLBCL by analysis of immunoglobulin variable region genes.

Previous studies from China and Singapore, another Asian country also with intermediate prevalence of HBV, have reported that 13.3% to 30.9% of DLBCL patients were HBsAg-positive, similar or even higher than the 13.8% in our study [17–20]. These results suggested that in HBV prevalent region, a certain percentage of DLBCL patients were chronically infected with HBV. We further found HBsAg-positive DLBCL patients showed unique clinical features, including an earlier disease onset age, a narrow range of onset age, a much more common involvement of spleen and retroperitoneal lymph nodes, and more advanced disease. An earlier disease onset age in HBsAg-positive patients was reported by studies from both Korea [3] and China [18, 19, 21]. A more advanced stage and frequent involvement of spleen in HBsAg-positive DLBCL patients were also reported by Wang et al. [18], but not by others [17, 19, 20, 22]. The discrepancy might be ascribed to the small sample sizes [17, 22], different prevalence of HBV infection, and inclusion of T-cell lymphoma which is less frequently associated with HBV infection [17, 23].

Most importantly, consistent with the results in two previous studies from China [18, 20, 21], our study showed HBsAg-positive DLBCL patients had a significantly poor outcome. Although the high risk factors associated with worse outcome, such as advanced disease, higher IPI and LDH (shown in Supplemental Table 1), were observed in HBsAg-positive DLBCL patients, they were related to the tumor biology itself but not caused by a biased patient selection because our cohort is a consecutive cohort. Further analysis showed that the poor outcome of HBsAg-positive DLBCL patients was mainly due to early PD. In our study, PD occurred in about 40% of the HBsAg-positive DLBCL patients, treated with or without rituximab. On the other hand, although HBsAg-positive DLBCL patients had a higher risk of developing hepatic dysfunction and HBV reactivation during anti-tumor therapy [13], the hepatic dysfunction higher than grade 2 and HBV reactivation both occurred in only 6.2% of HBsAg-positive DLBCL patients. All these complications were under control without causing death, thus contributed little to the poor outcome.

DLBCL can be stratified into GCB and non-GCB subtypes by immunohistochemistry, and patients with non-GCB subtype have an inferior prognosis [24]. We identified 67.3% in HBsAg-positive group and 56.9% in both HBsAg- and HBcAb-negative group as non-GCB subtype, without a significant difference between these two groups. In addition, for HBsAg-positive DLBCL patients, PFS and OS did not differ between the GCB and non-GCB subtypes. Thus, our study did not find a significant representation of cell of origin subtype in HBsAg-positive DLBCL, which may have an influence in the prognosis. However, due to the small sample size and without using the gold standard cell of origin classification by gene-expression profile, further studies on cell of origin in HBsAg-positive patients are needed.

Interestingly, our clinical data provided clues that these HBsAg-positive DLBCLs may result from chronic HBV-associated antigen stimulation based on three aspects. First, about 60% of the HBsAg-positive patients had a long history of chronic HBV infection; Secondly, the majority of HBsAg-positive DLBCL was developed between an age from 35 to 50, suggesting that the development of lymphoma needs a long time of chronic antigen stimulation; Thirdly, B-cells in spleen and retroperitoneal lymph nodes deal with antigen from blood circulation and from portal venous system respectively, which is consistent with the distribution of HBV-associated antigen. In fact, chronic antigen stimulation either as a result of infection or autoimmune disease, was commonly associated with some B-cell NHLs [25]. The two common clinical characteristics of these lymphomas were long-term history of infection or autoimmune diseases and specific involvement sites, which were also found in our HBV-associated DLBCL patients.

This hypothesis was substantiated by analysis of immunoglobulin variable region gene segments in subsets of HBsAg-positive patients. In our study, we first found a strongly biased usage of both Ig heavy and light chain genes in HBsAg-positive DLBCL, with individual *IGHV4-34* and *IGKV4-1* accounting for 42.1% and 65.5% respectively, both of which were much higher than that in normal peripheral blood B-cells and B-cell NHLs [26–30]. Of note, the *IGHV4-34* is known to play a role in viral infections and autoimmunity and *IGHV4-34* using B-cells entering to germinal center reaction suggests a striking antigen selection [31, 32]. Furthermore, more than two thirds of KCDR3 sequences were found to be highly stereotyped and the majority of them even were identical. Most importantly, almost all the HCDR3, KCDR3, and LCDR3 sequences exhibited a high homology to reported antibody sequences specific for HBV-associated antigen, mainly HBsAg. Lastly, most of these Ig genes were highly mutated.

In B-cell malignancies, the study of the antigen receptor variable region genes is a key tool to provide circumstantial evidence for both a possible involvement of antigen selection and their ontogenetic derivation. Different biased Ig gene usages and stereotyped CDR3 sequences suggesting antigen selective and ontogenetic processes were fully reported in many subtypes of B-cell malignancies [26, 29, 30, 33–35]. Furthermore, although the immunogenetic analysis in lymphoma had focused mainly on Ig heavy chain genes, a series of evidence support that Ig light chain might also play a substantial role in the antigen recognition [30, 33, 36, 37]. Consistent with these literatures, all the distinctive immunogenetic characteristics in our study strongly supported that chronic viral antigen stimulation might be an initiating event in the lymphomagenesis in chronic HBV-infected populations, although other mechanisms such as direct viral infection of B-cells need to be further investigated. This would be more plausible when the biologic features of HBV were taken into account. Chronic HBV infection is typically characterized by the production of extremely high quantities of viral proteins, and antibody responses are vigorous and sustained [38, 39].

The association between HBV and DLBCL would parallel that between HCV and NHL, where more extensive evidence regarding clinical features and pathogenesis mechanisms were available [10]. Till now, chronic viral antigen stimulation is the most plausible mechanism. The most frequent HCV-associated lymphoma subtypes originate from either germinal center or postgerminal center lymphocytes, suggesting lymphomagenesis occurs when B-cells proliferate in response to antigen. Further evidence comes from the observation that the B-cell receptor of HCV-associated lymphoma has biased usages of *VH1-69* and *VK3-27A* [40], as well as a homology of both IG heavy and light chain CDR3 sequence to anti-HCV E2 antibody [10]. However, apparent differences could be demonstrated between HCV-associated and HBV-associated B-cell lymphomas. First, HCV is a RNA virus but HBV a DNA virus, which definitely might result in different natural history, clinical consequences, and biologic features; secondly, except aggressive B-cell lymphoma, HCV-related B-cell lymphoma also include indolent lymphoma such as MZL and lymphoplasmacytic lymphoma, but our data showed that HBsAg-positive DLBCL was more aggressive and most of them had an extremely poor outcome.

Our study may provide useful information for the clinical management of both patients with HBV-associated DLBCL and the 350 million chronic HBV-infected populations. DLBCL is a highly heterogeneous disease. Based on their unique clinical features, poor outcome, and identical etiology, our study suggested that HBV-associated DLBCL might be considered as a unique subset of DLBCL. The standard R-CHOP therapy was far less enough for these patients. New therapeutic strategies such as treatment aiming to the B-cell receptor BCR signaling might be useful for these patients. Our study suggested that, unlike hepatocellular carcinoma (HCC) mainly occurs in patients with hepatitis B [16], B-cell lymphoma developed in patients with diverse manifestations of chronic HBV infections although mainly in chronic carriers. A routine screening of not only liver cirrhosis but also spleen and retroperitoneal masses in chronic HBV-infected population might help for an early diagnosis of HBV-associated lymphoma.

Our study had some limitations. The association between occult HBV-infection and B-cell NHL is not taken into account [12, 39]. In fact, there was a trend in our data that HBsAg-negative but HBcAb-positive DLBCL patients had worse survival compared with patients who were negative for both HBsAg and HBcAb. This result might be explained by the truth that HBcAb-positive populations were more likely to be occult HBV-infected patients and might develop HBV-associated DLBCL. Moreover, the KCDR3 sequence from an HBsAg-postive MCL patient also showed high homology to HBsAb (data not shown) suggesting other subtypes of B-cell lymphoma may also be related to HBV infection. These limitations may lead to an underestimation of the impact of HBV-infection on the development of lymphoma.

In conclusion, in conjunction with biologic and clinical information, our view of HBV-associated lymphoma may not only lead to a better understanding of the association between HBV and lymphoma, but also potentially pave the way for the development of new management strategies for both chronic HBV-infected patients and HBV-associated lymphoma.

## MATERIALS AND METHODS

### Patient selection and treatment

A total of 670 patients were consecutively diagnosed with DLBCL in Peking University Cancer Hospital from February 2001 to July 2010. The pathological diagnosis was confirmed according to the World Health Organization (WHO) Classification of Tumors of Hematopoietic and Lymphoid Tissues (2008). The staging was determined according to the Ann Arbor Staging Criteria. Eighty-three patients were excluded due to incomplete information and 587 patients were included in the study. All patients received a standard CHOP (cyclophosphamide, doxorubicin, vincristine, and prednisone) regimen or rituximab plus CHOP (R-CHOP) as the primary treatment. For 21 patients who have an active HBV infection with an HBV DNA level greater than 1 * 10^5^ IU/ml, rituximab was not used. Since June 2008, prophylactic antiviral therapy was given for 54 HBsAg-positive patients and 90 HBsAg-negative, HBcAb-positive patients up to 6 months after oncologic treatment ends, irrespectively of HBV-DNA level. The institutional ethics board approved the study.

### Immunohistochemistry and cell of origin classification

Immunohistochemical analysis was performed for CD10 (clone 56C6; Novocastra), BCL6 (clone LN22; Novocastra), and MUM1 (clone MUMp1; DAKO) using a streptavidin-biotin complex technique and 4-um thick unstained slides. The cell of origin classification was based on the Hans algorithm [24]. Cell of origin was identified in 49 of the 81 HBsAg-positive DLBCL patients. To exclude the impact of occult HBV infection, cell of origin was also identified in 93 of the 264 DLBCL patients who were negative for both HBsAg and HBcAb.

### HBV detection

Routine screening for viral markers including HBsAg, hepatitis-B surface antibody (HBsAb), hepatitis-B e antigen (HBeAg), hepatitis-B e antibody (HBeAb) and hepatitis-B core antibody (HBcAb) was performed by chemiluminescence immunoassay on Architect-i2000 (Abbott Laboratories). Real-time quantitative polymerase chain reaction was used to determine the HBV DNA copy number before chemotherapy if HBsAg or HBcAb is positive. All patients were also tested for serum human immunodeficiency virus (HIV) and hepatitis-C virus (HCV) antibody.

### Polymerase chain reaction (PCR) amplification

Total DNA was extracted from formalin-fixed paraffin-embedded tissue sections from 47 of the 81 HBsAg-positive DLBCL patients using the Omega DNA extraction kit (Omega, United States). Ig heavy chain, kappa and lambda chain gene segments from framework region 3 (FR3) were amplified according to the BIOMED-2 Concerted Action protocols [41]. For the samples with no detectable amplification of *IGHV-D-J* rearrangements from FR3, PCR was performed from framework region 2 (FR2). PCR products were sequenced directly, including both forward and reverse reads, using Bid-Dye terminators (Applied Biosystems, USA). All samples were tested in two separate PCR experiments and sequencing in both directions.

### Analysis of Ig heavy and light chain variable region

All obtained sequences were analyzed using the IMGT/V-QUEST database (http://www.imgt.org, last accessed August 6, 2014) [42]. We classified the sequences into truly unmutated, minimally/borderlinely mutated, and highly mutated groups as previously described [35]. We used previously described stringent criteria to define a stereotyped heavy and light chain complementarity determining region 3 (CDR3) amino acid sequence [36, 43]. Analyses were performed using the ClustalX 2.0 multiple sequence alignment software. The CDR3 sequences were also analyzed by the NCBI Basic Local Alignment Search Tool (BLAST) program (see http://blast.ncbi.nlm.nih.gov/Blast.cgi?form=0) for homology to multiple protein sequence databases [43].

### Statistical analysis

The groups of patients were compared by the Pearson's chi-square test or the Fisher exact test for categorical parameters, and Mann-Whitney U test for continuous variables. The differences were considered significant at *p* < 0.05. Overall survival (OS) was defined as the time elapsed from the date of diagnosis of DLBCL to the date of death or the last follow-up. Progression-free survival (PFS) was defined as the time elapsed between treatment initiation and tumor progression or death from any cause, with censoring of patients who are lost to follow-up. Survival curves were calculated by Kaplan-Meier method and the differences between two groups were compared by log-rank test. The multivariate analysis of outcome in terms of OS was performed by Cox regression, which included all the parameters with *p* ≤ 0.1 as determined by the univariate analysis. All calculations were made in SPSS/PC+ version 16.0.

## SUPPLEMENTARY FIGURE AND TABLE



## References

[1] Nowoslawski A, Brzosko WJ, Madalinski K, Krawczynski K (1970). Cellular localisation of Australia antigen in the liver of patients with lymphoproliferative disorders. Lancet.

[2] Heimann R, Ray MB, Desmet VJ (1977). HBsAg, chronic lymphoproliferative disorders, and cirrhosis of liver. Journal of clinical pathology.

[3] Kim JH, Bang YJ, Park BJ, Yoo T, Kim CW, Kim TY, Heo DS, Lee HS, Kim NK (2002). Hepatitis B virus infection and B-cell non-Hodgkin's lymphoma in a hepatitis B endemic area: a case-control study. Japanese journal of cancer research : Gann.

[4] Crook PD, Jones ME, Hall AJ (2003). Mortality of hepatitis B surface antigen-positive blood donors in England and Wales. International journal of epidemiology.

[5] Ulcickas Yood M, Quesenberry CP, Guo D, Caldwell C, Wells K, Shan J, Sanders L, Skovron ML, Iloeje U, Manos MM (2007). Incidence of non-Hodgkin's lymphoma among individuals with chronic hepatitis B virus infection. Hepatology.

[6] Engels EA, Cho ER, Jee SH (2010). Hepatitis B virus infection and risk of non-Hodgkin lymphoma in South Korea: a cohort study. The Lancet Oncology.

[7] Fwu CW, Chien YC, You SL, Nelson KE, Kirk GD, Kuo HS, Feinleib M, Chen CJ (2011). Hepatitis B virus infection and risk of intrahepatic cholangiocarcinoma and non-Hodgkin lymphoma: a cohort study of parous women in Taiwan. Hepatology.

[8] Nath A, Agarwal R, Malhotra P, Varma S (2010). Prevalence of hepatitis B virus infection in non-Hodgkin lymphoma: a systematic review and meta-analysis. Internal medicine journal.

[9] Dalia S, Chavez J, Castillo JJ, Sokol L (2013). Hepatitis B infection increases the risk of non-Hodgkin lymphoma: a meta-analysis of observational studies. Leukemia research.

[10] Marcucci F, Mele A (2011). Hepatitis viruses and non-Hodgkin lymphoma: epidemiology, mechanisms of tumorigenesis, and therapeutic opportunities. Blood.

[11] Vannata B, Zucca E (2014). Hepatitis C virus-associated B-cell non-Hodgkin lymphomas. Hematology / the Education Program of the American Society of Hematology American Society of Hematology Education Program.

[12] Marcucci F, Spada E, Mele A, Caserta CA, Pulsoni A (2012). The association of hepatitis B virus infection with B-cell non-Hodgkin lymphoma - a review. American journal of blood research.

[13] Kusumoto S, Tobinai K (2014). Screening for and management of hepatitis B virus reactivation in patients treated with anti-B-cell therapy. Hematology / the Education Program of the American Society of Hematology American Society of Hematology Education Program.

[14] Liang X, Bi S, Yang W, Wang L, Cui G, Cui F, Zhang Y, Liu J, Gong X, Chen Y, Wang F, Zheng H, Wang F, Guo J, Jia Z, Ma J (2013). Reprint of: Epidemiological serosurvey of Hepatitis B in China—declining HBV prevalence due to Hepatitis B vaccination. Vaccine.

[15] Lu FM, Zhuang H (2009). Management of hepatitis B in China. Chinese medical journal.

[16] European Association For The Study Of The L (2012). EASL clinical practice guidelines: Management of chronic hepatitis B virus infection. Journal of hepatology.

[17] Lim ST, Fei G, Quek R, Lim LC, Lee LH, Yap SP, Loong S, Tao M (2007). The relationship of hepatitis B virus infection and non-Hodgkin's lymphoma and its impact on clinical characteristics and prognosis. European journal of haematology.

[18] Wang F, Xu RH, Luo HY, Zhang DS, Jiang WQ, Huang HQ, Sun XF, Xia ZJ, Guan ZZ (2008). Clinical and prognostic analysis of hepatitis B virus infection in diffuse large B-cell lymphoma. BMC cancer.

[19] Xie W, Zhou D, Hu K, Xiao X, Huang W, He J, Shi J, Luo Y, Zhang J, Lin M, Cai Z, Huang H, Ye X (2013). Clinical analysis and prognostic significance of hepatitis B virus infections for diffuse large B-cell lymphoma with or without rituximab therapy. Experimental and therapeutic medicine.

[20] Wei Z, Zou S, Li F, Cheng Z, Li J, Wang J, Wang C, Chen F, Cao J, Cheng Y (2014). HBsAg is an independent prognostic factor in diffuse large B cell lymphoma patients in rituximab era: result from a multicenter retrospectie analysis in China. Medcine oncology.

[21] Wang F, Xu RH, Han B, Shi YX, Luo HY, Jiang WQ, Lin TY, Huang HQ, Xia ZJ, Guan ZZ (2007). High incidence of hepatitis B virus infection in B-cell subtype non-Hodgkin lymphoma compared with other cancers. Cancer.

[22] Law MF, Lai HK, Chan HN, Ha CY, Nq C, Yeung YM, Yip SF (2015). The impact of hepatitis B virus (HBV) infection on clinical outcomes of patients with diffuse large B-cell lymphoma. European Journal of Cancer Care.

[23] Chen J, Wang J, Yang J, Zhang W, Song X, Chen L (2013). Concurrent infection of hepatitis B virus negatively affects the clinical outcome and prognosis of patients with non-Hodgkin's lymphoma after chemotherapy. PloS one.

[24] Hans CP, Weisenburger DD, Greiner TC, Gascoyne RD, Delabie J, Ott G, Muller-Hermelink HK, Campo E, Braziel RM, Jaffe ES, Pan Z, Farinha P, Smith LM, Falini B, Banham AH, Rosenwald A (2004). Confirmation of the molecular classification of diffuse large B-cell lymphoma by immunohistochemistry using a tissue microarray. Blood.

[25] Zinzani PL (2012). The many faces of marginal zone lymphoma. Hematology / the Education Program of the American Society of Hematology American Society of Hematology Education Program.

[26] Bikos V, Darzentas N, Hadzidimitriou A, Davis Z, Hockley S, Traverse-Glehen A, Algara P, Santoro A, Gonzalez D, Mollejo M, Dagklis A, Gangemi F, Bosler DS, Bourikas G, Anagnostopoulos A, Tsaftaris A (2012). Over 30% of patients with splenic marginal zone lymphoma express the same immunoglobulin heavy variable gene: ontogenetic implications. Leukemia.

[27] Kraj P, Friedman DF, Stevenson F, Silberstein LE (1995). Evidence for the overexpression of the VH4–34 (VH4.21) Ig gene segment in the normal adult human peripheral blood B cell repertoire. Journal of immunology.

[28] Sebastian E, Alcoceba M, Balanzategui A, Marin L, Montes-Moreno S, Flores T, Gonzalez D, Sarasquete ME, Chillon MC, Puig N, Corral R, Pardal E, Martin A, Gonzalez-Barca E, Caballero MD, San Miguel JF (2012). Molecular characterization of immunoglobulin gene rearrangements in diffuse large B-cell lymphoma: antigen-driven origin and IGHV4-34 as a particular subgroup of the non-GCB subtype. The American journal of pathology.

[29] Baptista MJ, Calpe E, Fernandez E, Colomo L, Cardesa-Salzmann TM, Abrisqueta P, Bosch F, Crespo M (2014). Analysis of the IGHV region in Burkitt's lymphomas supports a germinal center origin and a role for superantigens in lymphomagenesis. Leukemia research.

[30] Smilevska T, Tsakou E, Hadzidimitriou A, Bikos V, Stavroyianni N, Laoutaris N, Fassas A, Alphanagnostopoulos A, Papadaki T, Belessi C, Stamatopoulos K (2008). Immunoglobulin kappa gene repertoire and somatic hypermutation patterns in follicular lymphoma. Blood cells, molecules & diseases.

[31] Mockridge CI, Rahman A, Buchan S, Hamblin T, Isenberg DA, Stevenson FK, Potter KN (2004). Common patterns of B cell perturbation and expanded V4-34 immunoglobulin gene usage in autoimmunity and infection. Autoimmunity.

[32] Pugh-Bernard AE, Silverman GJ, Cappione AJ, Villano ME, Ryan DH, Insel RA, Sanz I (2001). Regulation of inherently autoreactive VH4-34 B cells in the maintenance of human B cell tolerance. The Journal of clinical investigation.

[33] Bikos V, Stalika E, Baliakas P, Darzentas N, Davis Z, Traverse-Glehen A, Dagklis A, Kanellis G, Anagnostopoulos A, Tsaftaris A, Ponzoni M, Berger F, Felman P, Ghia P, Papadaki T, Oscier D (2012). Selection of antigen receptors in splenic marginal-zone lymphoma: further support from the analysis of the immunoglobulin light-chain gene repertoire. Leukemia.

[34] Zhu D, Lossos C, Chapman-Fredricks JR, Lossos IS (2013). Biased immunoglobulin light chain use in the Chlamydophila psittaci negative ocular adnexal marginal zone lymphomas. American journal of hematology.

[35] Hadzidimitriou A, Agathangelidis A, Darzentas N, Murray F, Delfau-Larue MH, Pedersen LB, Lopez AN, Dagklis A, Rombout P, Beldjord K, Kolstad A, Dreyling MH, Anagnostopoulos A, Tsaftaris A, Mavragani-Tsipidou P, Rosenwald A (2011). Is there a role for antigen selection in mantle cell lymphoma? Immunogenetic support from a series of 807 cases. Blood.

[36] Pighi C, Barbi S, Bertolaso A, Zamo A (2013). Mantle cell lymphoma cell lines show no evident immunoglobulin heavy chain stereotypy but frequent light chain stereotypy. Leukemia & lymphoma.

[37] Hadzidimitriou A, Darzentas N, Murray F, Smilevska T, Arvaniti E, Tresoldi C, Tsaftaris A, Laoutaris N, Anagnostopoulos A, Davi F, Ghia P, Rosenquist R, Stamatopoulos K, Belessi C (2009). Evidence for the significant role of immunoglobulin light chains in antigen recognition and selection in chronic lymphocytic leukemia. Blood.

[38] Ganem D, Prince AM (2004). Hepatitis B virus infection—natural history and clinical consequences. The New England journal of medicine.

[39] Chen MH, Hsiao LT, Chiou TJ, Liu JH, Gau JP, Teng HW, Wang WS, Chao TC, Yen CC, Chen PM (2008). High prevalence of occult hepatitis B virus infection in patients with B cell non-Hodgkin's lymphoma. Annals of hematology.

[40] Chan CH, Hadlock KG, Foung SK, Levy S (2001). V(H)1-69 gene is preferentially used by hepatitis C virus-associated B cell lymphomas and by normal B cells responding to the E2 viral antigen. Blood.

[41] Van Dongen JJ, Langerak AW, Bruggemann M, Evans PA, Hummel M, Lavender FL, Delabesse E, Davi F, Schuuring E, Garcia-Sanz R, van Krieken JH, Droese J, Gonzalez D, Bastard C, White HE, Spaargaren M (2003). Design and standardization of PCR primers and protocols for detection of clonal immunoglobulin and T-cell receptor gene recombinations in suspect lymphoproliferations: report of the BIOMED-2 Concerted Action BMH4-CT98-3936. Leukemia.

[42] Giudicelli V, Brochet X, Lefranc MP (2011). IMGT/V-QUEST: IMGT standardized analysis of the immunoglobulin (IG) and T cell receptor (TR) nucleotide sequences. Cold Spring Harbor protocols.

[43] De Re V, De Vita S, Marzotto A, Rupolo M, Gloghini A, Pivetta B, Gasparotto D, Carbone A, Boiocchi M (2000). Sequence analysis of the immunoglobulin antigen receptor of hepatitis C virus-associated non-Hodgkin lymphomas suggests that the malignant cells are derived from the rheumatoid factor-producing cells that occur mainly in type II cryoglobulinemia. Blood.

