# Supplementary material for: Hepatitis B virus-associated diffuse large B-cell lymphoma: unique clinical features, poor outcome, and hepatitis B surface antigen-driven origin
Source: Oncotarget. 2015 Jul 22;6(28):25061–73. doi: 10.18632/oncotarget.4677 (PMC4694815; doi:10.18632/oncotarget.4677)
Supplement: Supplementary file 1 [file oncotarget-06-25061-s001.pdf]

SUPPLEMENTARY FIGURE AND TABLE

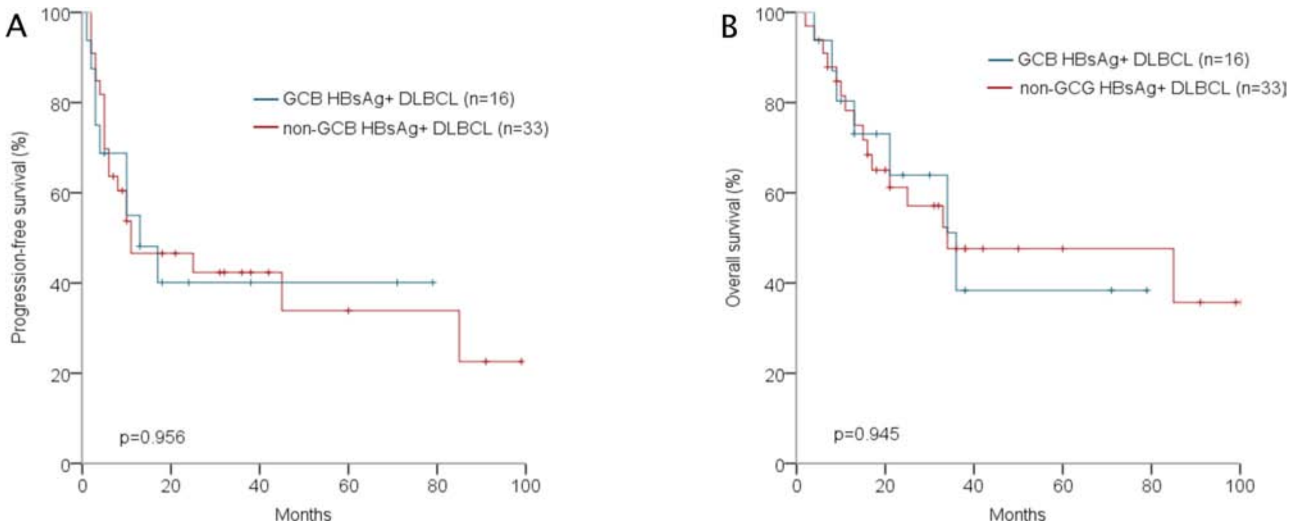

Supplementary Figure S1: Survival analysis according to cell of origin. Progression-free survival of HBsAg-positive DLBCL patients with GCB and non-GCB subtypes **A,** Overall survival of HBsAg-positive DLBCL patients with GCB and non-GCB subtypes **B.**

Supplementary Table S1: Univariate and multivariate analyses of clinical factors for OS rates of HBsAg-postitive DLBCL patients

Clinical factor	N (%)	OS			
		Univariate analysis		Multivariate analysis	
		HR (95% CI)	*P* value	HR (95% CI)	*P* value
Gender, male	51 (63.0)	1.19 (0.64–2.22)	0.589	ND	ND
Age > 60	12 (14.8)	1.62 (0.75–3.48)	0.222	ND	ND
Stage III/IV	62 (76.5)	4.52 (1.61–12.68)	**0.004**	2.70 (0.68–10.76)	0.158
Positive B symptom	47 (58.0)	2.51 (1.31–4.83)	**0.006**	1.47 (0.68–3.17)	0.329
PS 2–4	10 (12.3)	2.65 (1.22–5.75)	**0.014**	1.62 (0.62–4.27)	0.327
IPI 3–5	34 (42.0)	2.81 (1.54–5.13)	**0.001**	1.72 (0.53–5.59)	0.364
Elevated LDH	47 (58.0)	6.30 (2.89–13.74)	**<0.001**	5.31 (1.86–15.10)	**0.002**
Bulky mass	13 (16.0)	1.97 (0.94–4.13)	0.071	1.10 (0.46–2.61)	0.837
Extra-nodal sites ≥ 2	36 (44.4)	1.84 (1.02–3.34)	**0.044**	1.02 (0.35–2.97)	0.979
Without use of rituximab	33 (40.7)	1.76 (0.93–3.32)	0.083	1.29 (0.62–2.69)	0.502
Involvement of special sites					
Liver	9 (11.1)	2.00 (0.89–4.51)	0.095	1.15 (0.40–3.29)	0.797
Spleen	33 (40.7)	2.07 (1.14–3.76)	**0.017**	1.06 (0.53–2.09)	0.875
Retroperitoneal LN	50 (61.7)	3.11 (1.53–6.33)	**0.002**	1.12 (0.42–3.00)	0.821
Hepatic chirrosis	15 (18.5)	1.21 (0.58–2.52)	0.618	ND	ND
Hepatic dysfunction					
Before chemotherapy	19 (23.5)	1.63 (0.84–3.17)	0.151	ND	ND
During chemotherapy	30 (37.0)	2.13 (1.17–3.87)	**0.013**	ND	ND
Positive HBV-DNA	36 (44.4)	1.17 (0.57–2.42)	0.672	ND	ND
Without anti-virus therapy	54 (66.7)	1.56 (0.85–2.84)	0.738	ND	ND
Reactivation of HBV	7 (8.6)	1.02 (0.56–1.86)	0.954	ND	ND

Abbreviations: PS, performance status; IPI, International Prognostic Index; LDH, lactate dehydrogenase, LN, lymph node, HBV, hepatitis B virus, HR, hazard ratio; CI, confidence interval, ND, not done.
Bulky mass ≥ 10 cm.
